# Application of the FANTASTIC Lifestyle Questionnaire in the Academic Context

**DOI:** 10.3390/healthcare10122503

**Published:** 2022-12-10

**Authors:** Patrícia Batista, João Neves-Amado, Anabela Pereira, João Amado

**Affiliations:** 1 Human Neurobehavioral Laboratory (HNL), Research Centre for Human Development (CEDH), Universidade Católica Portuguesa, 4169-005 Porto, Portugal; 2 Instituto de Ciências da Saúde (ICS), Centro de Investigação Interdisciplinar em Saúde (CIIS), Universidade Católica Portuguesa, 4169-005 Porto, Portugal; 3 Centro de Investigação em Educação e Psicologia, Universidade de Évora, 7000-849 Évora, Portugal; 4 Centro de Investigação Interdisciplinar em Saúde (CIIS), Universidade Católica Portuguesa, 4169-005 Porto, Portugal

**Keywords:** FANTASTIC questionnaire, lifestyle, health behavior, university students, life quality

## Abstract

Studying citizens’ lifestyles is extremely important for understanding society and the social conditions of the personal lifestyle. Understanding the lifestyles of university students is especially important because they will be the future citizens and professionals who will shape society’s future. The aim of this study was a systematic review of the scientific literature about the use of the FANTASTIC Lifestyle questionnaire in an academic context. The reflective systematic literature review was carried out on PubMed, MEDLINE, Science Direct, and SCIELO databases with the keywords (“FANTASTIC Lifestyle questionnaire” OR “FANTASTICO questionnaire” OR “FANTASTIC questionnaire” OR “FANTASTIC survey” OR “FANTASTIC checklist”) AND (“university students”). The PRISMA criteria for reporting systematic reviews and meta-analyses were applied. The inclusion criteria were the use of the “FANTASTIC Lifestyle questionnaire” instrument for measuring lifestyles, the presentation of quantitative or qualitative results, and psychometric studies. The exclusion criteria were no use of FANTASTIC Lifestyle Questionnaire; other population, no quantitative or qualitative analysis, incomplete articles. The 15 scientific articles included in the study were analyzed. This literature review allowed us to conclude the importance and usefulness/actuality of this questionnaire. Furthermore, the importance of diagnosis should be emphasized, as should the development of strategies and intervention programs for the maintenance or creation of healthy societies.

## 1. Introduction

Lifestyles and health behaviors are key elements that condition the health of a society [1]. They determine a direct and/or indirect impact on health on the physical, mental, and social dimensions. Healthy lifestyles such as a healthy diet, regular physical activity, moderate alcohol consumption, and drug abstinence are factors that enhance citizens’ health and well-being. On the other hand, drug consumption (illegal drugs or tobacco and alcohol), malnutrition and an incorrect diet, sedentary behavior, and risky sexual behaviors are considered unhealthy lifestyles and are associated with the development and/or intensification of diseases, affecting the citizen’s health [2].

Therefore, it is important to diagnose lifestyles at all ages, especially at younger ages, for better intervention with promising results for the future of society. In this sense, it is important to develop promotion and intervention plans and strategies, as well as increase health literacy on this topic. The World Health Organization (WHO) highlights health literacy as a key element in the development of strategies to improve lifestyles [3,4].

Acting on younger populations and raising their awareness of the issue will help to promote health for future generations. In this sense, it is important to know the lifestyle of our young people today, the future professionals of tomorrow. It is important to make them aware of this issue, to increase their knowledge, and to adopt better practices in the future.

The academic environment is often an enabler of unhealthy lifestyles due to the many variables involved. University students are a critical group in terms of the adoption of risky health behaviors such as unhealthy diets, sedentary behavior, unhealthy sexual behavior, tobacco and alcohol consumption, poor (with reduced hours as well as the quality of) sleep, and others [4,5,6]. However, these young people are also more susceptible to change. If effective strategies are developed, they can not only encourage the development of healthy lifestyles but also be promoters of healthy lifestyles in the population. So, the university should be an active agent in health promotion [5]. The university has the responsibility to develop strategies (activities, programs) to promote and improve health and increase health literacy. The health-promoting practices are important in order to develop or increase healthy lifestyles in students and in future communities.

The FANTASTIC Lifestyle questionnaire was developed in 1983 by Wilson and collaborators to evaluate the population’s lifestyles and for use in a community health education program [7,8]. It is a simple questionnaire that can be completed quickly and evaluates nine dimensions. FANTASTIC is the acrostic that includes the dimensions: F = Family and friends (2 items); A = Activity and Associativity (3 items); N = Nutrition (3 items); T = Tobacco (2 items); A = Alcohol and other substances (6 items); S = Sleep and stress (3 items); T = Type of personality (3 items); I = Introspection (3 items); and C = Control of health (3 items).

Although it is a questionnaire used over several years, it is still used today because it is easy and accessible to fill out and because it can assess several dimensions [9]. Thus, this study aimed to know the use of this questionnaire in the university context (in university students), over time.

## 2. Methods

In this systematic literature review, studies were identified using a search of the databases in digital format, MEDLINE, PubMed, Science Direct, and SCIELO, between June 7 and August 25, 2022.

The keywords used in the research were: [(FANTASTIC Lifestyle questionnaire” OR “FANTASTICO questionnaire” OR “FANTASTIC questionnaire” OR “FANTASTIC survey” OR “FANTASTIC checklist”) AND (“University students”)]. The 33 articles found were then reduced to 15 following the removal of duplicates and those that did not meet the inclusion criteria. Other publications were discovered, but they were not indexed in these databases. The inclusion and exclusion criteria for the studies are described in Table 1.

**Figure 1:**
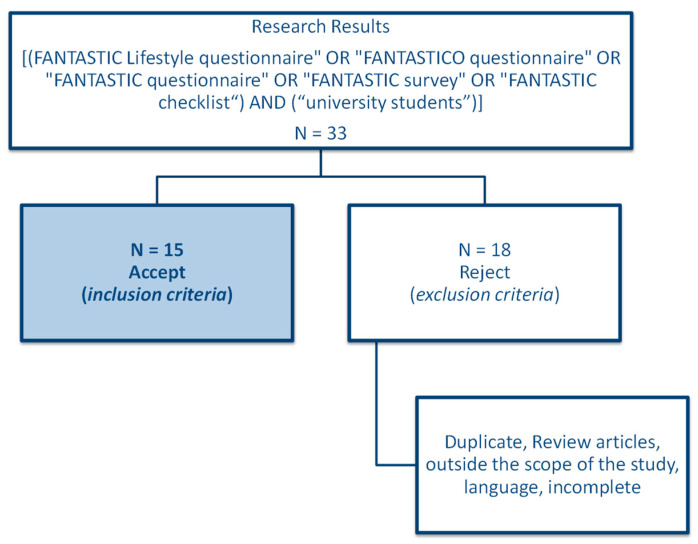
Flowchart applying the inclusion and exclusion criteria in research.

The procedure for conducting this systematic review was based on the PRISMA criteria, that is, preferred reporting items for systematic reviews and meta-analyses (Prisma)ª, were applied (Figure 1). The data were analyzed for several parameters, such as year of publication, authors, sample, country, type of study/methodology, results, and aim. The referencing was made through EndNote bibliographic referencing.

## 3. Results and Discussion

For this systematic review, 15 complete articles that met the inclusion criteria were analyzed. For a better understanding and analysis of the studies, a summary table was designed (Table 2). The table was created according to several elements: year of publication/authors, sample, country, methodology, instruments, domains of FANTASTIC, results, and aims.

The studies involved a total of 13,631 university students from Brazil, Colombia, Spain, Portugal, Chile, Poland, and Mexico. All studies used the FANTASTIC questionnaire; however, some studies evaluated all the dimensions of the questionnaire [5,6,10,11,12,13,14,15,16,17,18,19], while others only evaluated some dimensions, such as nutrition, consumption of alcohol, tobacco or drugs, physical activity, and sleep.

A cross-sectional study methodology was clearly distinguished, and two studies reported the instrument validation [9,18].

**Table 2:** Summary of the studies included in the analysis.

Author/Year	Sample	Country	Methodology	FLQ (Dimensions Evaluated)	Aim	Main Results
Murillo-Llorente et al., 2022 [9]	N = 501	Spain	Cross-sectional study	- Nutrition; - Tobacco; - Sleep.	- to validate the FANTASTIC questionnaire in a Spanish university population to report on the participants’ lifestyle.	- FANTASTIC questionnaire has good internal consistency (Cronbach’s Alpha = 0.797) and good construct validity (Kaiser–Meyer–Olkin value was 0.786).
Ruiz-Zaldibar et al., 2022 [5]	N = 488	Spain	Observational, descriptive, and cross-sectional survey study	- All dimensions	- to investigate the perceived changes in lifestyle behaviors among Spanish university students during COVID-19-related confinement.	- Students’ lifestyles worsened during the lockdown (female students were especially affected compared to their male peers (*p* = 0.010)). Female students change the “Good” standard of living for lower levels (classified as “Moderate,” “Low,” and “Worrying”); - Social/emotional behaviors were deeply affected, while confinement could be a protective factor against previous toxic habits.
Navarro-Cruz et al., 2021 [14]	N = 639	Chile	Cross-sectional study	- All dimensions	- to evaluate the association of differences in dietary behaviors and lifestyle with self-reported weight gain during the COVID-19 lockdown in Chile.	- Different dietary behaviors (mainly consumption of industrialized foods) during the lockdown, as well as quality-of-life deterioration, were the main factors associated with self-reported weight gain during the lockdown. Differences in lifestyle (odds ratio [OR] = 14.21); worsening eating habits (OR = 3.43).
Sousa et al., 2021 [19]	N = 150	Portugal	Cross-sectional study	- All dimensions	- to explore the mediation role of self-regulation on health-related behaviors adoption or maintenance, mental health, and well-being during the COVID-19 confinement in a sample of adults in Portugal.	- Self-regulation had direct effects on healthy habits and mental health, and indirect effects on well-being and mental health mediated by healthy habits; - Healthy habits exerted direct effects on well-being perception and mental health.
Gonçalves et al., 2020 [10]	N = 495	Portugal	Descriptive-correlational study	- All dimensions	- to analyze the propensity of young adults towards nomophobia and lifestyle.	- Positive and moderate correlation between nomophobia and psychopathological symptoms (Somatization (r = 0.322), Obsession-Compulsion (r = 0.394), Interpersonal Sensitivity (r = 0.390), Depression (r = 0.374), Anxiety (r = 0.340), Hostility (r = 0.384), Paranoid Ideation (r = 0.381), and Psychoticism (r = 0.382); *p* < 0.001.
Machul et al., 2020 [12]	N = 444	Poland	Cross-sectional study	- All dimensions	- to analyze the lifestyle practices, life satisfaction, and level of perceived stress of Polish and foreign students.	- Polish students obtained higher (mean = 36.26 ± 6.21) results in FLQ, and stress levels than foreign students (mean = 33.55 ± 6.71); - The self-assessment of their health condition, lifestyle, and rank associated with being healthy correlated with the FLQ, Satisfaction with Life Scale, and Perceived Stress Scale.
González-Cantero et al., 2017 [11]	N = 320	Mexico	Cross-sectional and correlational study	- All dimensions	- to determine the relationship between the psychological capital (CapPsi) and lifestyle of Mexican university students.	- CapPsi improves lifestyle; resilience (r = 0.505, *p* < 0.01); hope (r = 0.432, *p* < 0.01); optimism (r = 0.412, *p* < 0.01); and self-efficacy (r = 0.400, *p* < 0.01). The CapPsi (R2 = 0.32) explained 33.3 percent of the total variance of the lifestyle. However, further research is necessary to determine the CapPsi influence on the adoption and/or maintenance of a healthy lifestyle.
Martinez-Torres et al., 2017 [13]	N = 890	Colombia	Cross-sectional study	- All dimensions	- to investigate the prevalence and the associated variables of the Metabolic syndrome (MetS) in Colombian collegiate students.	- The prevalence of MetS was 6.0%, and it was higher in men than women; - The predisposing factors for having a MetS include being male, over 23 years old, overweight or obese, and having an unhealthy waist-to-height ratio.
Ramírez-Vélez et al., 2017 [20]	N = 1687	Colombia	Cross-sectional study	- Alcohol; - Tobacco; - Physical activity.	- to investigate body fat percentage and fat mass index thresholds for the prediction of metabolic syndrome in students.	- Based on the International Diabetes Federation criteria, both index thresholds seem to be good tools to identify university students with unfavorable metabolic profiles.
Tassini et al., 2017 [6]	N = 57	Brazil	Descriptive, cross-sectional, population study	- All dimensions	- to compare the factors determining the quality of life of students in the healthcare area using the FLQ.	- The overall rating was “regular”, and none of the participants scored in the “very good” or “excellent” categories. - Domains requiring change include nutrition and physical activity among medical students and cigarette, drug, and alcohol consumption among physical therapy students.
Rodríguez-Gázquez et al., 2016 [17]	N = 380	Colombia	Cross-sectional study	- All dimensions	- to assess the lifestyles of nursing students at a Colombian public university.	- An important proportion of university students have inadequate lifestyles (poor in 9.2%, fair in 31.3%), which means deferred risks for the development of chronic diseases.
Ramírez-Vélez et al., 2015 [16]	N = 5921	Colombia	Cross-sectional study	-All dimensions	- to assess the lifestyle of a sample of university students.	- “good lifestyle” was perceived by 57.4% of females and 58.5% of males; - Despite the students being evaluated for referring to themselves as having a healthy lifestyle, stated behavior involving a health risk were observed in the domains concerning nutrition, physical activity, and smoking.
Pacheco et al. 2014 [15]	N = 716	Brazil	Cross-sectional study	- All dimensions	- to determine the association between lifestyle and sociodemographic variables of freshmen attending a state university in southern Brazil.	- Inadequate lifestyle prevalence (5.3%). - Adjusted analysis results indicated that students over 20 years old, whose mothers’ formal education lasted less than nine years had a higher risk of having an inadequate lifestyle.
Silva et al., 2014 [18]	N = 707	Portugal	Cross-sectional study	- All dimensions	- to translate, culturally adapt, and validate the FLQ in a group of higher education students in Portugal	- The instrument demonstrated good overall internal consistency for an instrument used to measure a latent variable; - The construct validity tested by the instrument’s classification capacity in four, three, and two categories was 67.6%, 67.6%, and 100%, respectively, with a Kappa index of 0.55, 0.55, and 1.00; - The FLQ is a reliable and valid instrument for assessing young adults’ lifestyles.
Ferrari et al., 2013 [21]	N = 236	Brazil	Descriptive and cross-sectional study	- Tobacco; - Alcohol and drugs; - Nutrition.	- to determine the prevalence of and factors associated with body image dissatisfaction among physical education students enrolled in a public university.	- The prevalence of body image dissatisfaction was 69.5%; 44.1% were dissatisfied with excess weight; - A Body Mass Index ≥ 25.0 kg/m^2^ was associated with dissatisfaction with excess weight; - Factors associated with dissatisfaction with slimness were being male, eating an unhealthy diet, and smoking tobacco.

This literature review has shown that despite having been developed in 1983 [7], the FANTASTIC questionnaire is still in use today [22,23]. This instrument has been used in several research areas with different target groups and countries. Recently, during the pandemic, it was used in different studies to diagnose or evaluate the lifestyles of different populations, in particular university students [5,19,22,24,25].

It is a simple and user-friendly instrument that is easy to fill in and easy to interpret and analyze. On the other hand, it is a complete instrument that can be used to diagnose and evaluate several dimensions, as mentioned before. This questionnaire is flexible and can still be used in different contexts and with different target groups, but in the present study, we were concerned to know its use in the university context, more specifically its application to university students, as shown in Table 2.

Based on the literature, we found that this questionnaire has been used in several studies (see Table 2). This topic is very relevant, since the university environment, the transition to a new life, new friends, and being away from family are some of the factors that lead to a change in university students’ lifestyles. This behavior modification can be beneficial in some cases or can potentiate the acquisition of risky behaviors. Therefore, studying the students’ lifestyles makes perfect sense to understand reality and may help develop strategies to promote healthy practices.

Most of the studies analyzed aimed to evaluate the various dimensions of lifestyle present in the instrument [5,6,10,11,12,13,14,15,16,17,18,19]. However, some studies are concerned with evaluating individual lifestyle components, such as diet, and their importance and relation to pathologies [9,21]. Stress, physical activity, sleep, tobacco, alcohol, and drug consumption are other dimensions that have also been highlighted [9,20,21].

Therefore, knowing the perception of university students’ lifestyles is fundamental to designing health promotion actions and strategies for intervention. The university has a leading role in the complete education of students, academically, socially, and for a healthy society, considering the sustainable development goals. The education of these students, the acquisition of personal skills, and the increase in health literacy can contribute to the development of healthier future generations. The adoption of healthy lifestyles will improve quality of life, promote health, and disease prevention. The WHO has been calling attention to this situation, highlighting the role of healthy lifestyles in health and disease prevention [16,26,27,28]. However, this is a multidimensional and interdisciplinary challenge that involves various dimensions such as environmental, social, economic, governmental, legal, and interpersonal influences on individuals’ health and behavior [12,29]. For a healthy society, governments or health entities should adopt diagnosis and intervention strategies. These strategies should be outlined from the beginning of the youth’s education. Increased health literacy is fundamental to the adoption of healthy lifestyles in society and the improvement of people’s quality of life and health. So, it is necessary to develop interventions mainly aimed at the creation or enhancement of healthy behaviors and toward the reduction of those behaviors that are health risks [30]. Therefore, the data obtained from a survey of students could be used as a basis for providing health coaching, health information, and student literacy related to specific lifestyle-related, changeable health risks.

This literature review draws attention to the relevance of this topic and the studies that have been developed to date. The outcome of this work is information on the utility of the FANTASTIC tool and current patterns of use in assessing university-age individuals. In this sense, the use of the FANTASTIC Lifestyle questionnaire could become a pre-diagnosis instrument and a preventative tool to evaluate specific risk behaviors in several populations, in particular the university student population. Knowing and improving the lifestyle diagnosis of these young people will enable the design of more effective health promotion strategies. Encouraging wider use of the tool based on the systematic review might be a better-stated outcome.

## 4. Limitations and Implications for Practice

One possible limitation in this study could be the methodology selected, which could have conditioned the results obtained. The search strategies, the database, and the keywords selected may have been restrictive and may have left out many valid data studies.

Although the FANTASTIC questionnaire was the specific goal of this study, the limitation to a single instrument of lifestyle evaluation may condition students’ perceptions of lifestyle studies in light of world reality.

This literature review allowed us to find studies that reported the use of the FANTASTIC questionnaire in the diagnosis of the lifestyles of university students. Thus, the relevance and appropriateness of using this questionnaire are also highlighted, as is the need for further studies.

Its use enables not only the identification and diagnosis of these students’ lifestyles but also the improvement of their health literacy. On the other hand, it will allow universities to develop health promotion strategies for these age groups, with an impact on the students themselves and the community.

## 5. Conclusions

Lifestyle is considered one of the major determinants of individual and collective health. The university context should be a place of academic and personal education, enabling the acquisition of personal skills that allow the adoption of healthy lifestyles. In this way, universities should develop actions to diagnose lifestyles, enhance health literacy, and promote healthy lifestyles.

The use of diagnostic tools such as the FANTASTIC questionnaire should be a strategy to promote health. This systematic review intended to draw attention to the importance of healthy lifestyles and their diagnosis through the use of a FANTASTIC questionnaire, one that was simple, comprehensive, and easy to access, which allowed not only diagnosis but also increased health literacy on this topic. This questionnaire has been used over time and is of great importance for the analysis of the various components involved in the evaluation of lifestyle. Evaluation to encourage changes that promote health and well-being is one of the outcomes of this work.

## Figures and Tables

**Table 1:** Criteria for inclusion and exclusion from the study.

*Inclusion criteria*	*Exclusion criteria*
- Use of FANTASTIC Lifestyle Questionnaire	- No use of FANTASTIC Lifestyle Questionnaire
- University students	- Other population
- Quantitative or Qualitative Analysis	- No Quantitative or Qualitative Analysis
- Psychometric studies (validation of scale/instrument)	- Incomplete articles
- Full articles	
- Language: English, Brazilian, Spanish and Portuguese	
